# Metacercarial Infection of Wild Nile Tilapia (*Oreochromis niloticus*) from Brazil

**DOI:** 10.1155/2014/807492

**Published:** 2014-11-19

**Authors:** Hudson A. Pinto, Vitor L. T. Mati, Alan L. Melo

**Affiliations:** Laboratório de Taxonomia e Biologia de Invertebrados, Departamento de Parasitologia, Instituto de Ciências Biológicas, Universidade Federal de Minas Gerais, P.O. Box 486, 30123970 Belo Horizonte, MG, Brazil

## Abstract

Fingerlings of *Oreochromis niloticus* collected in an artificial urban lake from Belo Horizonte, Minas Gerais, Brazil, were evaluated for natural infection with trematodes. Morphological taxonomic identification of four fluke species was performed in *O. niloticus* examined, and the total prevalence of metacercariae was 60.7% (37/61). *Centrocestus formosanus*, a heterophyid found in the gills, was the species with the highest prevalence and mean intensity of infection (31.1% and 3.42 (1–42), resp.), followed by the diplostomid *Austrodiplostomum compactum* (29.5% and 1.27 (1-2)) recovered from the eyes. Metacercariae of *Drepanocephalus* sp. and *Ribeiroia* sp., both found in the oral cavity of the fish, were verified at low prevalences (8.2% and 1.6%, resp.) and intensities of infection (only one metacercaria of each of these species per fish). These species of trematodes are reported for the first time in *O. niloticus* from South America. The potential of occurrence of these parasites in tilapia farming and the control strategies are briefly discussed.

## 1. Introduction

The Nile tilapia,* Oreochromis niloticus* (Linnaeus, 1758), is a cichlid species native to Africa that, due to characteristics such as rapid reproduction rate and growth, omnivorous feeding, and hardiness, has been used in aquaculture in different parts of the world including America, Africa, and Asia. In fact, tilapia are the second most important fish group of farmed fish, after carps, with a global aquaculture production of more than 3 millions of tonnes in 2012 [1]. On the other hand, the intentional or accidental introduction of* O. niloticus* in urban aquatic environments has been reported. In these places, nonnative cichlids can contribute to the processes of eutrophication caused mainly by phosphorus excretion and resulting from bioturbation due to their bottom-feeding habits, causing increase in total phosphorus and chlorophyll a concentrations as well as high cyanobacteria densities [2]. Moreover, introduction of* O. niloticus* may be related to environmental impacts as competition with other aquatic species, including native fish species [3, 4]. Given the current economic importance of* O. niloticus* worldwide, studies on its parasite fauna are desirable in order to prevent economic losses. In this context, a diversity of protozoan and metazoan species has been reported in these hosts (both wildlife and captive) in tropical and subtropical countries [5–11].

In Brazil,* O. niloticus* was introduced in the 1970s, and currently it is the main fish used in fish farming [12]. This invasive alien species is now established in a great number of Brazilian reservoirs, where it has achieved high population densities and become the predominant species, which is common in sportive and subsistence fishing in the country [13–15]. Records on the natural infection of this invasive cichlid with Crustacea, Monogenea, and Protozoa species are recent in the country and in an increasing number (reviewed by [8]). However, reports of infection of* O. niloticus* with trematodes are comparatively scarce. In fact, the only report of trematodes in* O. niloticus* from Brazil was performed by Silva et al. [16], who found metacercariae identified as* Clinostomum complanatum* Rudolphi, 1814, a causative agent of the Yellow Grub Disease.

In the present study, new reports of trematodes in* O. niloticus* are presented based on parasitological analyses of wild specimens collected in an urban reservoir from Brazil. Moreover, the potential of occurrence of these parasites in tilapia farming is discussed.

## 2. Material and Methods

Sixty-one specimens of* O. niloticus*, fingerlings, nonsexed, measuring 2–4.5 cm in total length, and weighing 0.15–1.49 g, were collected in the Pampulha reservoir (19°50′18′′S; 43°59′40′′W), an artificial lake located in Belo Horizonte, Minas Gerais, Brazil, in June 2013. Fish were caught with the aid of a D-shaped nylon hand net (50 cm wide, 40 cm high with a 30 cm opening and 1 mm^2^ mesh) and transported to the laboratory. Then they were measured, weighed, and killed by means of cerebral concussion (in accordance with the procedures recommended by the local ethics committee on animal experimentation, CETEA, UFMG) and examined for the presence of parasites. Initially, the organs and tissues (gills, intestine, skin, and musculature) were separated in Petri dishes containing saline (0.85% NaCl) and dissected with the aid of metal needles under a stereomicroscope. The gill arches were separated and transferred to glass slides and examined under a light microscope. After this preliminary examination, the fish tissues were subjected to artificial digestion by 1% pepsin solution in 0.85% NaCl and 1% HCl (pH 2) for 1 hour at 37°C and reanalyzed using a stereoscopic microscope.

The trematodes recovered were subjected to morphological study in light microscope. Photographs were taken on a Leica microscope coupled with a Leica ICC50 HD digital camera. Measurements were performed with the aid of a micrometer eyepiece. Taxonomic identification, until the lowest possible category, was carried out according to different authors [17–21]. The specimens were deposited in the collection of the Department of Parasitology (DPIC) at Federal University of Minas Gerais, Brazil. The ecological terms were used according to Bush et al. [22].

## 3. Results

From 61 specimens of* O. niloticus* evaluated, 37 (60.7%) were found infected with metacercariae. A total of 94 metacercariae were recovered and four species of trematodes were identified. The species identified are listed below and the measures (micrometers, as the mean followed by the standard deviation and the range in parentheses) are presented in Table 1.

**Figure 1:**
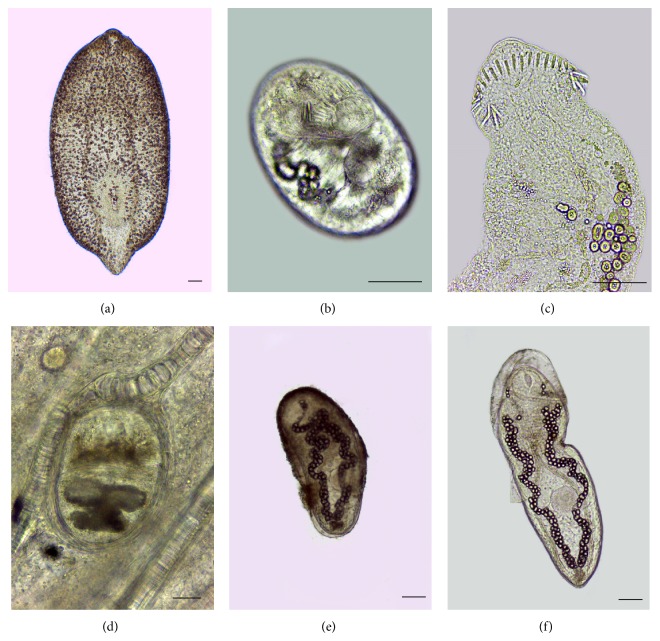
Metacercariae found in naturally infected specimens of Nile tilapia (*Oreochromis niloticus*) collected in Pampulha reservoir, Belo Horizonte, Minas Gerais, Brazil. (a)* Austrodiplostomum compactum* recovered in the eyes; (b) encysted* Drepanocephalus* sp. found in oral cavity; (c) mechanically excysted specimen of* Drepanocephalus* sp. and detail of spines of cephalic collar; (d)* Centrocestus formosanus* found encysted in the gills; (e)* Ribeiroia* sp. recovered encysted in oral cavity; and (f) mechanically excysted specimen of* Ribeiroia* sp. All images obtained from unstained wet mount preparation. Scale bar = 50 *μ*m.

*Austrodiplostomum compactum (Lutz, 1928) (Diplostomidae) (Figure 1(a))*

 Site of infection: eyes.
 Prevalence of infection: 29.5% (18/61).
 Mean intensity of infection: 1.27 (1-2).
 Mean abundance of infection: 0.41.

*Remarks*. This diplostomid species is an intestinal parasite of cormorants (*Phalacrocorax* spp.) in the American continent [23–26]. In Brazil, metacercariae of* A. compactum* were reported in about forty native fish species (reviewed by [27]). Nevertheless, it had not yet been reported in* O. niloticus* in the country. Adult parasites were found in* Phalacrocorax brasilianus* (Gmelin, 1789) in Brazil [26] and snails of the genus* Biomphalaria* Preston, 1910, are the first intermediate hosts [20]. The infection of* O. niloticus* with* A. compactum*, including reports of the occurrence of mortality, was previously reported in Mexico [28] and Panama [29].

*Centrocestus formosanus (Nishigori, 1924) (Heterophyidae) (Figure 1(d))*

 Site of infection: gill filaments.
 Prevalence of infection: 31.1% (19/61).
 Mean intensity of infection: 3.42 (1–42).
 Mean abundance of infection: 1.03.

*Remarks*.* Centrocestus formosanus* is an intestinal heterophyid of Asian origin reported in birds and mammals, including human [18]. The formation of metacercariae in gills is related to the occurrence of asphyxia and mortality as well as delayed development, which cause damage to fish farming [30]. In Brazil,* C. formosanus* was recorded firstly in its molluscan intermediary host, the invader thiarid* Melanoides tuberculata* (Müller, 1774) [31, 32], and recently reported in fish (*Australoheros facetus* (Jenyns, 1842) and* Poecilia reticulata* Peters, 1859) and bird (*Butorides striata* (Linnaeus, 1758)) from the Pampulha reservoir [19, 33, 34]. This heterophyid was previously reported in* O. niloticus* from Egypt [35, 36], Vietnam [37–39], and Saudi Arabia [40] but had not yet been reported in this cichlid in South America. In addition, the occurrence of mortality in* O. niloticus* experimentally infected with* C. formosanus* was verified in Costa Rica [41].

*Drepanocephalus sp. (Echinostomatidae) (Figures 1(b)-1(c))*

 Site of infection: oral cavity and musculature.
 Prevalence of infection: 8.2% (5/61).
 Intensity of infection: 1.
 Mean abundance of infection: 0.082.

*Remarks*. This 27-collar spined echinostomatid species parasitizes cormorants (*Phalacrocorax* spp.) in the Americas. Two species,* Drepanocephalus spathans* Dietz, 1909, and* Drepanocephalus olivaceus* Nasir and Marval, 1968, were reported in* P. brasilianus* from Brazil [17, 26]. The life cycle of a species of this genus was recently elucidated and involves planorbid snails,* Planorbella trivolvis* (Say, 1817) (=*Helisoma trivolvis*) in North America [42]. In South America,* Biomphalaria* spp. are probably the first intermediate hosts of* Drepanocephalus* spp. The metacercariae of* Drepanocephalus* sp. recovered in* O. niloticus* have spines of the cephalic collar with general morphology and disposition compatible with those reported in adult parasites [16]. Metacercariae of* Drepanocephalus* spp. were reported in fish from Mexico [43–46] and recently in the USA [42]. This is the first report of metacercariae of* Drepanocephalus* in fish from South America. The potential association between the infection by these echinostomids with mortality and delayed development of North American fish was discussed [42].

*Ribeiroia sp. (Psilostomidae) (Figures 1(e)-1(f))*

 Site of infection: oral cavity.
 Prevalence of infection: 1.6% (1/61).
 Intensity of infection: 1.
 Mean abundance of infection: 0.016.

*Remarks*. The trematodes of the genus* Ribeiroia* Travassos, 1939 are proventricular parasites of birds (with a few reports in rodents) from the Americas and Africa. In Brazil,* Ribeiroia insignis* Travassos, 1939, considered by some authors as a synonymous of* Ribeiroia ondatrae* (Price, 1931), was recorded in cormorants and herons [17, 26], and the participation of the planorbid* B. straminea* in transmission of* Ribeiroia* was recently reported [47]. These parasites have currently acquired great importance since* R. ondatrae* is involved in malformation and mortality of amphibians in North America [48], a phenomenon not yet reported in South American hosts. Moreover the natural infection of 15 species of fish, including* O. niloticus*, with* R. ondatrae* or* Ribeiroia marini* (Faust and Hoffman, 1934) was reported in North and Central America by a few authors (reviewed by [48]). To date, fish were not reported harboring metacercariae of* Ribeiroia* in South America. Studies in order to determine other fish species involved in the transmission of* Ribeiroia* spp. and the potential pathological effects of these parasites in these hosts are lacking. Preliminary experimental infection studies revealed the occurrence of cutaneous alterations in fish due* Ribeiroia* sp. from Brazil (unpublished results), likely a result of the intense inflammatory process during the initial phase of infection [49]. 

Most of the infected fish were found harboring one species of trematodes (31/37 specimens, 83.8%). Coinfection was verified in 16.2% (6/37) of the parasitized fish, in which 4 specimens of* O. niloticus* were found infected with* C. formosanus* and* A. compactum*, 1 specimen with* A. compactum* and* Drepanocephalus* sp., and 1 specimen with* A. compactum* and* Ribeiroia* sp.

## 4. Discussion

The four species of trematodes found in* O. niloticus* in the present study are generalists regarding their second intermediate hosts. In fact,* A. compactum* have been reported in several species of native fish from Brazil [27] while* Drepanocephalus* spp. and* Ribeiroia* sp. were reported in different species of freshwater fish from North America [44–46, 48]. Given the wide distribution of species belonging to these three genera in their respective definitive hosts, mainly cormorants of the genus* Phalachrocorax*, both parasites will surely be found in* O. niloticus* in other localities from South America. The same statement is valid for* C. formosanus,* an invasive alien species that has been recorded widely in new locations and fish species in the American continent.

Prevalence and intensity of infection with trematodes may be influenced by factors such as age of fish, behavior, and resistance to parasitism. In fact, the prevalence and intensity of infection by* C. formosanus* observed in* O. niloticus* in the Pampulha reservoir are smaller than those previously verified for* A. facetus*, a native cichlid, from the same area of study (100%, 134 metacercariae/fish resp.) [19]. On the other hand, low intensities of infection for* C. formosanus* and* A. compactum* were also reported in* O. niloticus* by different authors from other countries [35–40], which may suggest a relative resistance of this fish to these parasites. Indeed, resistance to parasites is a reason for the cultivation of tilapia; however, regarding trematodes, this protection is not absolute, and so these parasites can be related to the occurrence of potential losses in the tilapia farming.

The fact that the sample evaluated was composed of fingerling may also be related to low values of intensity of infection observed in the present study. Nevertheless, the prevalence of infection is considerable in the case of fingerling. These two ecological data and also the diversity of parasites may be higher in adult fish from the Pampulha reservoir, probably due to longer exposure to parasitic infective stages, as verified for other parasites species found in* O. niloticus* from Uganda [7]. Furthermore, considering* Drepanocephalus* sp. and* Ribeiroia* sp., species only now reported in fish from Brazil, factors such as the small size and location of the metacercariae in the host (adhered to oral cavity and musculature) may have made it difficult to detect the parasites in previous studies performed in the country. Thus, the artificial digestion of host tissues, which is not routinely used in studies of parasite fauna of fish may have increased the sensitivity to the detection of these metacercariae.

The trematodes found in* O. niloticus* in the present study are transmitted by molluscs,* M. tuberculata*, the intermediate host of* C. formosanus*, and* Biomphalaria* spp., the transmitters of* Drepanocephalus* sp.,* Ribeiroia* sp., and* A. compactum*. These mollusc species are widely distributed within watersheds in South America, and they are likely involved in the transmission of these parasites to* O. niloticus*, both wild and cultivated. The presence of these first intermediate hosts, associated with the occurrence of potential definitive hosts, mainly aquatic birds, may favor the introduction and maintenance of life cycle of trematodes in these places. In this sense, monitoring the occurrence of aquatic molluscs in extensive and semi-intensive tilapia farming is necessary for the prevention of outbreaks caused by these and other trematodes having fish, including* O. niloticus*, as second intermediate hosts. Among more than 20 species of digenetic trematodes infecting* O. niloticus* (mainly in Africa), metacercariae of species belonging to the genera* Apharyngostrigea*,* Clinostomum*,* Euclinostomum*,* Prohemistomum*, and* Tylodelphis* [5, 6, 50, 51] have been reported in vertebrate definitive hosts or native fish from Brazil [17, 52]. In addition to the possible effects on fish production, it is necessary to pay attention to species with zoonotic potential, as* C. formosanus* and* Clinostomum* spp., which have already been reported infecting humans in Asia.

Taxonomic studies have revealed a wide diversity of parasite species in fish, including* O. niloticus*, in Brazil [8, 52–55] and control measures have been discussed mainly in relation to parasite with direct transmission (Protozoa, Monogenea, and Crustacea). However, the potential impacts of trematode species in Brazilian fish farming are neglected and factors related to parasitism and pathological changes caused by trematodes in fish, including mortality, are dependent on several variables related to the parasite biology and host intrinsic factors [55]. Although the infection with adult digeneans often does not cause significant pathological changes in the fish hosts, metacercariae of several species such as diplostomids and heterophyids, when present in high parasite burden, are known to cause developmental delay or death, and so damage to pisciculture. In fact, it is estimated that one of the species found in the present study,* C. formosanus*, is the cause of losses of 3.5 million of dollars to pisciculture annually in the USA [30] and diplostomid species, including* A. compactum*, may be associated with mortality in catfish farming [56]. Accordingly, some strategies for the monitoring and control of these trematodes and their transmitter molluscs have been tested in North America [30, 56–58].

In South America, and especially in Brazil, the impact of trematodes in fish farming is still unknown, and the knowledge related to different fish-host susceptibility, prophylaxis, and control of these complex life cycle parasites needs advance. Thus, in the field of Brazilian tilapia production in extensive and semi-intensive conditions, the presence of aquatic molluscs and potential definitive hosts (aquatic birds such as cormorants, herons, and grebes) should serve as a warning about the possibility of maintaining the life cycle of species of trematodes potentially involved in occurrence of damage to fish farming.

## Figures and Tables

**Table 1:** Measurements of metacercariae of four species of trematodes found in naturally infected specimens of Nile tilapia (*Oreochromis niloticus*) collected in Pampulha reservoir, Belo Horizonte, Minas Gerais, Brazil.

		*Austrodiplostomum compactum *	*Centrocestus formosanus *	*Drepanocephalus* sp.	*Ribeiroia *sp.
Cyst	*L*	—	220 ± 11 (191–239)	193 ± 7 (186–200)	432
	*W*	—	145 ± 10 (123–171)	125 ± 3 (121–128)	205
Free/excysted metacercaria	*L*	1,464 ± 70 (1,375–1,581)	—	—	623
	*W*	702 ± 59 (602–808)	—	—	201
Oral sucker	*L*	68 ± 7 (61–82)	—	—	85
	*W*	70 ± 6 (61–82)	—	—	83
Ventral sucker	*L*	NP	—	—	75
	*W*	NP	—	—	77
Tribocytic organ	*L*	345 ± 22 (300–375)	NP	NP	NP
	*W*	180 ± 17 (164–212)	NP	NP	NP

*L* = length, *W* = width, and NP = not present.

## References

[1] Food and Agriculture Organization of the United Nations (FAO), Cultured Aquatic Species Information Programme http://www.fao.org/fishery/culturedspecies/Oreochromis_niloticus/en.

[2] Starling F., Lazzaro X., Cavalcanti C., Moreira R. (2002). Contribution of omnivorous tilapia to eutrophication of a shallow tropical reservoir: evidence from a fish kill. *Freshwater Biology*.

[3] Canonico G. C., Arthington A., Mccrary J. K., Thieme M. L. (2005). The effects of introduced tilapias on native biodiversity. *Aquatic Conservation: Marine and Freshwater Ecosystems*.

[4] Vicente I. S. T., Fonseca-Alves C. E. (2013). Impact of introduced Nile tilapia (*Oreochromis niloticus*) on non-native aquatic ecosystems. *Pakistan Journal of Biological Sciences*.

[5] Landsberg J. H., Shilo M., Sarig S. (1989). Parasites and associated diseases of fish in warm water culture with special emphasis on intensification. *Fish Culture in Warm Water Systems: Problems and Trends*.

[6] Florio D., Gustinelli A., Caffara M., Turci F., Quaglio F., Konecny R. (2009). Veterinary and public health aspects in tilapia *(Oreochromis niloticus niloticus*) aquaculture in Kenya, Uganda and Ethiopia. *Ittiopatologia*.

[7] Akoll P., Konecny R., Mwanja W. W., Nattabi J. K., Agoe C., Schiemer F. (2012). Parasite fauna of farmed Nile tilapia (*Oreochromis niloticus*) and African catfish (*Clarias gariepinus*) in Uganda. *Parasitology Research*.

[8] Pantoja W. M. F., Neves L. R., Dias M. K. R., Marinho R. G. B., Montagner D., Tavares-Dias M. (2012). Protozoan and metazoan parasites of Nile tilapia *Oreochromis niloticus* cultured in Brazil. *Revista MVZ Córdoba*.

[9] Ashade O. O., Osineye O. M., Kumoye E. A. (2013). Isolation, identification and prevalence of parasites on *Oreochromis niloticus* from three selected river systems. *Journal of Fisheries and Aquatic Science*.

[10] Wiriya B., Clausen J. H., Inpankaew T., Thaenkham U., Jittapalapong S., Satapornvanit K., Dalsgaard A. (2013). Fish-borne trematodes in cultured Nile tilapia (*Oreochromis niloticus*) and wild-caught fish from Thailand. *Veterinary Parasitology*.

[11] Penprapai N., Chumchareon M. (2013). Biodiversity of parasites in red tilapia fishes (*Oreochromis niloticus* Linn.) cultured cage in Trang River at Trang Province. *Journal of Applied Sciences Research*.

[12] Ministério da Pesca e Aquicultura (2011). *Boletim Estatístico da Pesca e Aquicultura*.

[13] Walter T., Petrere M. (2007). The small-scale urban reservoir fisheries of Lago Paranoá, Brasília, DF, Brazil. *Brazilian Journal of Biology*.

[14] Novaes J. L. C., Carvalho E. D. (2011). Artisanal fisheries in a Brazilian hypereutrophic reservoir: Barra Bonita reservoir, middle Tietê river. *Brazilian Journal of Biology*.

[15] Novaes J. L. C., Carvalho E. D. (2013). Analysis of artisanal fisheries in two reservoirs of the upper Paraná River basin (Southeastern Brazil). *Neotropical Ichthyology*.

[16] Silva A. S., Monteiro S. G., Doyle R. L., Pedron F. A., Filipetto J. E. S., Neto J. R. (2008). Ocorrência de *Clinostomum complanatum* em diferentes espécies de peixes de uma piscultura do município de Santa Maria—RS. *Veterinária e Zootecnia*.

[17] Travassos L., Freitas J. F. T., Kohn A. (1969). Trematódeos do Brasil. *Memórias do Instituto Oswaldo Cruz*.

[18] Scholz T., Salgado-Maldonado G. (2000). The introduction and dispersal of *Centrocestus formosanus* (Nishigori, 1924) (Digenea: Heterophyidae) in Mexico: a review. *The American Midland Naturalist*.

[19] Pinto H. A., Melo A. L. (2012). Metacercariae of *Centrocestus formosanus* (Trematoda: Heterophyidae) in *Australoheros facetus* (Pisces: Cichlidae) in Brazil. *Brazilian Journal of Veterinary Parasitology*.

[20] Pinto H. A., Melo A. L. (2013). *Biomphalaria straminea* and *Biomphalaria glabrata* (Mollusca: Planorbidae) as new intermediate hosts of the fish eyefluke *Austrodiplostomum compactum* (Trematoda: Diplostomidae) in Brazil. *Journal of Parasitology*.

[21] Beaver P. C. (1939). The morphology and life history of *Psilostomum ondatrae* Price, 1931 (Trematoda: Psilostomidae). *Journal of Parasitology*.

[22] Bush A. O., Lafferty K. D., Lotz J. M., Shostak A. W. (1997). Parasitology meets ecology on its own terms: Margolis et al. revisited. *Journal of Parasitology*.

[23] Lutz A., Lutz A. (1928). Estudios sobre trematodes observados en Venezuela. *Estúdios de Zoologia y Parasitologia Venezolanas*.

[24] Ostrowski de Núñez M. (1982). Die Entwicklungszyklen von *Diplostomum* (*Austrodiplostomum*) *compactum* (Lutz, 1928) und *D.* (*A.*) *mordax* (Szidat und Nani, 1951) n. comb in Südamerika. *Zoologischer Anzeiger*.

[25] Violante-González J., Monks S., Gil-Guerrero S., Rojas-Herrera A., Flores-Garza R., Larumbe-Morán E. (2011). Parasite communities of the neotropical cormorant *Phalacrocorax brasilianus* (Gmelin) (Aves, Phalacrocoracidae) from two coastal lagoons in Guerrero state, Mexico. *Parasitology Research*.

[26] Monteiro C. M., Amato J. F. R., Amato S. B. (2011). Helminth parasitism in the Neotropical cormorant, *Phalacrocorax brasilianus*, in Southern Brazil: effect of host size, weight, sex, and maturity state. *Parasitology Research*.

[27] Ramos I. P., Franceschini L., Zago A. C., Zica É. D. O. P., Wunderlich A. C., Carvalho E. D., da Silva R. J. (2013). New host records and a checklist of fishes infected with *Austrodiplostomum compactum* (Digenea: Diplostomidae) in Brazil. *Brazilian Journal of Veterinary Parasitology*.

[28] Violante-González J., García-Varela M., Rojas-Herrera A., Guerrero S. G. (2009). Diplostomiasis in cultured and wild tilapia *Oreochromis niloticus* in Guerrero State, Mexico. *Parasitology Research*.

[29] Roche D. G., Leung B., Franco E. F. M., Torchin M. E. (2010). Higher parasite richness, abundance and impact in native versus introduced cichlid fishes. *International Journal for Parasitology*.

[30] Mitchell J., Overstreet R. M., Goodwin A. E., Brandt T. M. (2005). Spread of an exotic fish-gill trematode: a far-reaching and complex problem. *Fisheries*.

[31] Pinto H. A., Melo A. L. (2010). *Melanoides tuberculata* (mollusca: Thiaridae) as an intermediate host of *Centrocestus formosanus* (Trematoda: Heterophyidae) in Brazil. *Revista do Instituto de Medicina Tropical de Sao Paulo*.

[32] Paula-Andrade C., Pinto H. A., Coscarelli D., Vidigal T. H. D. A., Melo A. L. (2012). The natural infection of *Melanoides tuberculata* (Müller, 1774) (Mollusca: Gastropoda) by *Centrocestus formosanus* (Nishigori, 1924) (Platyhelminthes: Trematoda) in Paranoá lake, Brasília, Brazil. *Brazilian Journal of Biology*.

[33] Pinto H. A., Melo A. L. (2012). Infecção natural de *Poecilia reticulata* (Actinopterygii: Poeciliidae) por metacercárias na Represa da Pampulha, Belo Horizonte, Minas Gerais, Brasil. *Boletim do Instituto de Pesca*.

[34] Pinto H. A., Mati V. L. T., Melo A. L. (2013). New records and a checklist of trematodes from *Butorides striata* (Aves: Ardeidae). *Revista Mexicana de Biodiversidad*.

[35] Ramadan R. A. M., Saleh O. A., El-Gamal R. M. (2002). Prevalence and distribution of metacercariae of *Centrocestus* sp. (Trematode: Heterophyidae) on gills and other organs of *Oreochromis niloticus* fingerlings. *Suez Canal Veterinary Medicine Journal*.

[36] Eissa A. M., Derwa H. I., Ismail M., Ramadan R. A., Zaki M., Mohamed N. (2014). Use of enzyme activities as biomarkers for oxidative stress induced by metacercarial affections in some cultured tilapia species. *Life Science Journal*.

[37] Arthur J. R., Te B. Q. (2006). Checklist of the parasites of fishes of Viet Nam. *FAO Fisheries Technical Paper no.*.

[38] Hop N. T., De N. V., Murrell D., Dalsgaard A. (2007). Occurrence and species distribution of fishborne zoonotic trematodes in wastewater-fed aquaculture in northern Vietnam. *Tropical Medicine and International Health*.

[39] Chi T. T. K., Dalsgaard A., Turnbull J. F., Tuan P. A., Murrell K. D. (2008). Prevalence of zoonotic trematodes in fish from a Vietnamese fish-farming community. *Journal of Parasitology*.

[40] Kalantan A. M. N., Al-Harbi A. H., Arfin M. (1999). On the metacercaria of *Centrocestus formosanus* (Trematoda:Heterophyidate) Nishigori, 1924 (Digenea:Heterophyidae) from *Oreochromis niloticus* in Saudi Arabia and its development in various definitive hosts. *Journal of Parasitology and Applied Animal Biology*.

[41] Cortés D. A., Dolz G., Zúñiga J. J. R., Jiménez A. E., Léon D. (2010). *Centrocestus formosanus* (Opisthorchiida: Heterophyidae) como causa de muerte de alevines de tilapia gris *Oreochromis niloticus* (Perciforme: Cichlidae) en el Pacífico seco de Costa Rica. *Revista de Biología Tropical*.

[42] Griffin M. J., Khoo L. H., Quiniou S. M., O'Hear M. M., Pote L. M., Greenway T. E., Wise D. J. (2012). Genetic sequence data identifies the cercaria of *Drepanocephalus spathans* (Digenea: Echinostomatidae), a parasite of the double-crested cormorant (*Phalacrocorax auritus*), with notes on its pathology in juvenile channel catfish (*Ictalurus punctatus*). *Journal of Parasitology*.

[43] Jiménez-García I. (1993). Fauna helmintológica de *Cichlasoma fenestratum* (Pisces: Cichlidae) del lago de Catemaco, Veracruz, México. *Anales del Instituto de Biologia, Universidad Nacional Autónoma de México, Serie Zoología*.

[44] Scholz T., Aguirre-Macedo M. L., Salgado-Maldonado G., García-Aldrete A. N., Vidal-Martinez V. M. (2000). Metacercariae of trematodes parasitizing freshwater fish in Mexico: a reappraisal and methods of study. *Metazoan Parasites in the Neotropic: Ecological, Taxonomic and Evolutionary Perspectives*.

[45] Salgado-Maldonado G. (2006). Checklist of helminth parasites of freshwater fishes from Mexico. *Zootaxa*.

[46] Pérez-Ponce de León G., García-Prieto L., Mendoza-Garfias B. (2007). Trematode parasites (Platyhelminthes) of wildlife vertebrates in Mexico. *Zootaxa*.

[47] Pinto H. A., Jadin R. C., Orlofske S. A., Johnson P. T. J., Melo A. L. (2013). *Biomphalaria straminea* (Mollusca: Planorbidae) as an intermediate host of *Ribeiroia* sp. (Trematoda: Psilostomidae) in Brazil. *Journal of Parasitology*.

[48] Johnson P. T. J., Sutherland D. R., Kinsella J. M., Lunde K. B. (2004). Review of the trematode genus *Ribeiroia* (Psilostomidae): ecology, life history and pathogenesis with special emphasis on the amphibian malformation problem. *Advances in Parasitology*.

[49] Huizinga H. W., Nadakavukaren M. J. (1997). Cellular responses of goldfish, *Carassius auratus* (L.), to metacercariae of *Ribeiroia marini* (Faust & Hoffman, 1934). *Journal of Fish Diseases*.

[50] Taher A. (2009). Some studies on metacercarial infection in *Oreochromis niloticus* in Assiut Governorate and their role in transmission of some trematodes to dogs. *Assiut University Bulletin for Environmental Researches*.

[51] Gustinelli A., Caffara M., Florio D., Otachi E. O., Wathuta E. M., Fioravanti M. L. (2010). First description of the adult stage of *Clinostomum cutaneum* Paperna, 1964 (Digenea: Clinostomidae) from grey herons *Ardea cinerea* L. and a redescription of the metacercaria from the Nile tilapia *Oreochromis niloticus niloticus* (L.) in Kenya. *Systematic Parasitology*.

[52] Eiras J. C., Takemoto R. M., Pavanelli G. C. (2010). *Diversidade dos parasitos de peixes de água doce do Brasil*.

[53] Eiras J. C., Takemoto R. M., Pavanelli G. C., Adriano E. A. (2011). About the biodiversity of parasites of freshwater fish from Brazil. *Bulletin of the European Association of Fish Pathologists*.

[54] Kohn A., Fernandes B. M. M., Cohen S. C. (2007). *South Americam Trematodes Parasites of Fishes*.

[55] Thatcher V. E. (2006). *Amazon Fish Parasites*.

[56] Overstreet R. M., Curran S. S. (2004). Defeating diplostomoid dangers in USA catfish aquaculture. *Folia Parasitologica*.

[57] Harvey M., Wagner E., Wilson C. (2005). Potential impact on June sucker, and control of the digenetic trematode *Centrocestus formosanus*, and its intermediate host, *Melanoides tuberculata*. *Final Report to the Utah Reclamation Mitigation and Conservation Commission no.*.

[58] Cantu V., Brandt T. M., Arsuffi T. L. (2013). An evaluation of three sampling methods to monitor a digenetic trematode *Centrocestus formosanus* in a spring-fed ecosystem. *Parasitology*.

